# Influenza vaccination might reduce the risk of ischemic stroke in patients with atrial fibrillation: A population-based cohort study

**DOI:** 10.18632/oncotarget.22352

**Published:** 2017-11-09

**Authors:** Pai-Feng Kao, Ju-Chi Liu, Yi-Ping Hsu, Li-Chin Sung, Tsung-Yeh Yang, Wen-Rui Hao, Ying-Chin Lin, Szu-Yuan Wu

**Affiliations:** ^1^ Division of Cardiovascular Medicine, Department of Internal Medicine, Shuang Ho Hospital, Taipei Medical University, New Taipei City, Taiwan; ^2^ Department of Family Medicine, Shung Ho Hospital, Taipei Medical University, New Taipei, Taiwan; ^3^ Department of Family Medicine, School of Medicine, College of Medicine, Taipei Medical University, Taipei, Taiwan; ^4^ Institute of Toxicology, College of Medicine, National Taiwan University, Taipei, Taiwan; ^5^ Department of Radiation Oncology, Wan Fang Hospital, Taipei Medical University, Taipei, Taiwan; ^6^ Department of Internal Medicine, School of Medicine, College of Medicine, Taipei Medical University, Taipei, Taiwan; ^7^ Department of Biotechnology, Hungkuang University, Taichung, Taiwan

**Keywords:** influenza vaccination, atrial fibrillation, ischemic stroke

## Abstract

**Purpose:**

Atrial fibrillation (AF) is associated with the risk of ischemic stroke, regardless of the administration of appropriate antithrombotic prophylaxis. This study investigated whether influenza vaccination is associated with the risk of ischemic stroke, to determine a solution to reduce this risk in patients with AF.

**Methods:**

We used data from the Taiwan National Health Insurance Research Database. The study cohort comprised all patients diagnosed as having AF (n = 14 454) before January 1, 2005; these patients were followed until December 31, 2012. The index date was January 1, 2005. A propensity score was derived using a logistic regression model to estimate the effect of vaccination by accounting for covariates that predict receiving the intervention (vaccine). A Cox proportional hazard model was used to calculate the hazard ratios (HRs) of ischemic stroke in vaccinated and unvaccinated patients with AF.

**Results:**

We included 6570 patients (2547 [38.77%] with and 4023 [61.23%] without influenza vaccination). The adjusted HRs (aHRs) of ischemic stroke were lower in the vaccinated patients than in the unvaccinated patients (influenza season, noninfluenza season, and all seasons: aHRs = 0.59, 0.50, and 0.55; *P* < 0.001, *P* < 0.001, and *P* < 0.001, respectively).

**Conclusions:**

Influenza vaccination might exert a dose-response effect against ischemic stroke in patients with AF who have risk factors for ischemic stroke by reducing the incidence of ischemic stroke, particularly in those aged 65–74 and ≥75 y.

## INTRODUCTION

Ischemic stroke may be a presenting manifestation of atrial fibrillation (AF) in some patients and may occur in some patients despite the administration of appropriate antithrombotic prophylaxis [1–3]. Compared with emboli from carotid disease, AF has been strongly associated with more severe ischemic stroke and relatively long-term transient ischemic attack, presumably because of embolization of large particles in AF [4, 5]. Moreover, AF is associated with particularly severe ischemic stroke mostly caused by relatively large emboli from the left atrial appendage [6].

The efficacy of antithrombotic therapy in preventing recurrent stroke in patients with AF and ischemic stroke has been extensively reported [7–10]. Anticoagulant therapy effectively reduced the risk of systemic embolization in patients with AF [10]. However, anticoagulant therapy may increase the risk of major bleeding [11]. The CHADS2 score is a clinical prediction rule for estimating stroke risk in patients with AF, a common and severe form of congestive heart failure associated with ischemic stroke [12]. This score is used to determine whether patients should receive anticoagulation or antiplatelet therapy, because AF can cause blood stasis in the upper heart chambers, leading to the formation of a mural thrombus that can dislodge into blood flow, reach the brain, interrupt the blood supply to the brain, and cause stroke [12]. Because the risk of major bleeding is increased in most patients receiving anticoagulants, careful consideration of the risk-to-benefit ratio is necessary.

An alternative treatment can be beneficial for patients with AF having a risk of ischemic stroke and can reduce the risk of major bleeding in patients receiving anticoagulants. Lavallee et al. and Nichol et al. have suggested that influenza vaccination in elderly patients aged 60–65 y prevents brain infarction by reducing infections [13, 14]. Taken together, these findings indicate that AF is associated with a high risk of ischemic stroke, regardless of the administration of appropriate antithrombotic prophylaxis, and that anticoagulant use increases the risk of major bleeding in most patients with AF.

Influenza infection can activate systemic inflammatory responses and increase the sympathetic tone that plays a crucial role in the pathogenesis of AF [15, 16]. In Taiwan, influenza infection was significantly associated with the development of AF and significantly increased the risk of AF, which could be reduced through influenza vaccination [17]. Theoretically, reducing AF risk or administering influenza vaccine might reduce the risk of ischemic stroke. The current study investigated the association of influenza vaccination with the risk of ischemic stroke to determine a solution to reduce the risk of ischemic stroke in patients with AF.

## RESULTS

The study cohort consisted of 6570 patients, of whom 2547 (38.77%) and 4023 (61.23%) received and did not receive influenza vaccination, respectively (Table 1). The total follow-up durations of the unvaccinated and vaccinated patients were 12 291.9 and 14 810.0 person-y, respectively. The prevalence of preexisting medical comorbidities, namely dyslipidemia (*P* = 0.003), vascular disease (*P* < 0.001), pneumonia (*P* < 0.001), and dialysis (*P* = 0.009), was higher in the unvaccinated patients than in the vaccinated patients. By contrast, the prevalence of congestive heart failure (*P* < 0.001) was higher in the vaccinated patients than in the unvaccinated patients. In addition, the distribution of age, monthly income, urbanization level, and drug use significantly differed between the vaccinated and unvaccinated patients (Table 1). A higher proportion of the vaccinated patients used warfarin, statin, metformin, ACEI, and aspirin for >28 cDDDs [18]. A lower proportion of the vaccinated patients had a monthly income of ≥NT$33,301 and resided in urban areas. Table 2 presents the risk of ischemic stroke observed in the unvaccinated and vaccinated patients. We calculated PSs after adjusting for age, sex, CCI, comorbidities, urbanization level, and monthly income. The adjusted HRs (aHRs) of ischemic stroke were lower in the vaccinated patients than in the unvaccinated patients (influenza season, noninfluenza season, and all seasons: aHRs = 0.59, 0.50, and 0.55; *P* < 0.001, *P* < 0.001, and *P* < 0.001, respectively). The stratified analysis revealed that the aHRs remained significantly lower in the vaccinated patients, particularly in those aged 65–74 and ≥75 y, regardless of sex. The aHRs of ischemic stroke were lower in the vaccinated patients than in the unvaccinated patients during all seasons (aged 55–64, 65–74, and ≥75 y: aHRs = 0.69, 0.48, and 0.45; *P* < 0.05, *P* < 0.001, and *P* < 0.001, respectively). During the influenza or noninfluenza season, the aHRs decreased regardless of age or sex, except in the 55–64-y-old age group, which had a relatively smaller sample size compared with the other subgroups (Table 2). Notably, despite the small sample size of this age group, the aHRs remained significantly lower in the vaccinated patients during the noninfluenza season. The stratified analysis indicated that the aHRs were significantly lower in the vaccinated patients, irrespective of sex, age, or season. The aHRs of ischemic stroke were lower in the vaccinated patients than in the unvaccinated patients during all seasons (women and men: aHRs = 0.51 and 0.61; *P* < 0.001 and *P* < 0.001, respectively). The aHRs remained significantly lower in the vaccinated patients during the influenza season, particularly in the women. Moreover, the aHRs were significantly lower in the vaccinated men during the noninfluenza season.

**Table 1:** Characteristics of the sample population

	Whole cohort (n =6570)		Unvaccinated patients (n = 4023)		Vaccinated patients (n = 2547)		*P*^a^
	n	%	n	%	n	%	
Age, y (Mean ± SD)	73.39 (9.65)		72.79 (10.68)		74.33 (7.67)		<0.001
55–64	1502	22.86	1188	29.53	314	12.33	<0.001
65–74	2111	32.13	1059	26.32	1052	41.30	
≥75	2957	45.01	1776	44.15	1181	46.37	
Sex							
Women	3100	47.18	1913	47.55	1187	46.60	0.453
Men	3470	52.82	2110	52.45	1360	53.40	
CCI							
0	1004	15.28	596	14.81	408	16.02	0.007
1	1346	20.49	812	20.18	534	20.97	
2	1386	21.10	814	20.23	572	22.46	
≥3	2834	43.14	1801	44.77	1033	40.56	
Comorbidities							
Diabetes	2244	34.16	1408	35.00	836	32.82	0.070
Hypertension	4922	74.92	2983	74.15	1939	76.13	0.071
Dyslipidemia	2550	38.81	1618	40.22	932	36.59	0.003
Congestive heart failure	3108	47.31	1810	44.99	1298	50.96	<0.001
Vascular disease	1010	15.37	686	17.05	324	12.72	<0.001
Pneumonia	1436	21.86	971	24.14	465	18.26	<0.001
Dialysis	452	6.88	303	7.53	149	5.85	0.009
Warfarin							
<28 cDDD	5113	77.82	3225	80.16	1888	74.13	<0.001
≥28 cDDD	1457	22.18	798	19.84	659	25.87	
Aspirin							
<28 cDDD	2422	36.86	1698	42.21	724	28.43	<0.001
≥28 cDDD	4148	63.14	2325	57.79	1823	71.57	
Statin							
<28 cDDD	4775	72.68	3040	75.57	1735	68.12	<0.001
≥28 cDDD	1795	27.32	983	24.43	812	31.88	
ACEI							
<28 cDDD	2163	32.92	1580	39.27	583	22.89	<0.001
≥28 cDDD	4407	67.08	2443	60.73	1964	77.11	
Metformin							
<28 cDDD	5249	79.89	3292	81.83	1957	76.84	<0.001
≥28 cDDD	1321	20.11	731	18.17	590	23.16	
Urbanization level							
Urban	4287	65.25	2736	68.01	1551	60.90	<0.001
Suburban	1546	23.53	893	22.20	653	25.64	
Rural	737	11.22	394	9.79	343	13.47	
Monthly income (NT$)							
0	840	12.79	509	12.65	331	13.00	<.001
1–21 000	1704	25.94	936	23.27	768	30.15	
21 000–33 300	2535	38.58	1495	37.16	1040	40.83	
≥33 301	1491	22.69	1083	26.92	408	16.02	

^a^Comparison between the unvaccinated and vaccinated patients

Vascular disease (e.g., peripheral artery disease, myocardial infarction, or aortic plaque)

**Table 2:** Risk of ischemic stroke in unvaccinated and vaccinated patients

All Group (n = 6570)	Unvaccinated patients (Total follow-up duration: 12 291.9 person-y)		Vaccinated patients (Total follow-up duration: 14 810.0 person-y)		aHR^†^ (95% CI)
	No. of patients with stroke	Incidence Rate (per 1000 person-y) (95% CI)	No. of patients with stroke	Incidence rate (per 1000 person-y) (95% CI)	
**Whole cohort**					
Influenza season	286	2326.7 (2057.1, 2596.4)	284	1917.6 (1694.6, 2140.7)	0.59 (0.50, 0.71)^***^
Noninfluenza season	222	1806.1 (1568.5, 2043.6)	174	1174.9 (1000.3, 1349.5)	0.50 (0.40, 0.61)^***^
All seasons	508	4132.8 (3773.4, 4492.2)	458	3092.5 (2809.3, 3375.7)	0.55 (0.48, 0.63)^***^
**Age, 55–64 y^a^**					
Influenza season	60	1200.5 (896.7, 1504.3)	31	1286.8 (833.8, 1739.8)	0.80 (0.51, 1.25)
Noninfluenza season	42	840.4 (586.2, 1094.5)	12	498.1 (216.3, 780.0)	0.52 (0.27, 1.00)^*^
All seasons	102	2040.9 (1644.8, 2436.9)	43	1785.0 (1251.4, 2318.5)	0.69 (0.48, 0.99)^*^
**Age, 65–74 y^b^**					
Influenza season	75	2217.8 (1715.9, 2719.8)	121	1793.6 (1474.0, 2113.2)	0.55 (0.41, 0.75)^***^
Noninfluenza season	70	2070.0 (1585.0, 2554.9)	71	1052.5 (807.6, 1297.3)	0.41 (0.29, 0.57)^***^
All seasons	145	4287.8 (3589.9, 4985.7)	192	2846.1 (2443.5, 3248.7)	0.48 (0.39, 0.61)^***^
**Age, ≥75 y^c^**					
Influenza season	151	3859.6 (3244.0, 4475.2)	132	2334.3 (1936.0, 2732.5)	0.49 (0.39, 0.63)^***^
Noninfluenza season	110	2811.6 (2286.2, 3337.1)	91	1609.2 (1278.6, 1939.9)	0.47 (0.35, 0.62)^***^
All seasons	261	6671.3 (5861.9, 7480.6)	223	3943.5 (3425.9, 4461.1)	0.45 (0.40, 0.53)^***^
**Women^d^**					
Influenza season	153	2625.7 (2209.6, 3041.7)	122	1772.1 (1457.6, 2086.5)	0.49 (0.38, 0.63)^***^
Noninfluenza season	107	1836.2 (1488.3, 2184.2)	85	1234.6 (972.2, 1497.1)	0.53 (0.39, 0.72)^***^
All seasons	260	4461.9 (3919.5, 5004.3)	207	3006.7 (2597.1, 3416.3)	0.51 (0.42, 0.61)^***^
**Men^e^**					
Influenza season	133	2057.3 (1707.7, 2407.0)	162	2044.1 (1729.3, 2358.8)	0.74 (0.58, 0.95)^*^
Noninfluenza season	115	1778.9 (1453.8, 2104.0)	89	1123.0 (889.7, 1356.3)	0.46 (0.34, 0.62)^***^
All seasons	248	3836.2 (3358.8, 4313.7)	251	3167.0 (2775.2, 3558.8)	0.61 (0.50, 0.74)^***^

^*^P < 0.05 ^**^P < 0.01 ^***^P < 0.001

^a^Total follow-up durations: 4997.9 and 2409.0 person-y for unvaccinated and vaccinated patients, respectively.

^b^Total follow-up durations: 3381.7 and 6746.1 person-y for unvaccinated and vaccinated patients, respectively.

^c^Total follow-up durations: 3912.3 and 5654.9 person-y for unvaccinated and vaccinated patients, respectively.

^d^Total follow-up durations: 5827.1 and 6884.6 person-y for unvaccinated and vaccinated patients, respectively.

^e^Total follow-up durations: 6464.7 and 7925.4 person-y for unvaccinated and vaccinated patients, respectively.

CI: confidence interval

HR: hazard ratio

aHR: adjusted hazard ratio

^†^The main model was adjusted for age; sex; Charlson comorbidity index; comorbidities of diabetes, hypertension, dyslipidemia, congestive heart failure, vascular disease, pneumonia, and dialysis; urbanization level; and monthly income by using propensity scores.

In the sensitivity analysis, adjustments were made to examine the association of age, sex, CCI, comorbidities, urbanization level, monthly income, and drug use with the incidence of ischemic stroke in different models. As presented in Table 3, the effects of vaccination remained significant in the subgroups of various covariates during the influenza season. Vaccination dose-dependently reduced the risk of ischemic stroke in all the subgroups and the main model with additional covariates (warfarin, statin, metformin, ACEI, or aspirin use). All the aHRs indicated that vaccination significantly reduced the risk of ischemic stroke in all the subgroups, regardless of comorbidities or drug use (*P* < 0.001). Our data revealed that the vaccination frequency reflected a protective effect against ischemic stroke during the influenza season. The protective effect was more predominant in the patients aged ≥75 y (1 vaccination: aHR = 0.71, 95% confidence interval [CI]: 0.52, 0.97; 2 or 3 vaccinations: aHR = 0.45, 95% CI: 0.31, 0.63; and ≥4 vaccinations: aHR = 0.35, 95% CI: 0.24, 0.51) and in those with diabetes (≥4 vaccinations: aHR = 0.37, 95% CI: 0.23, 0.59), CCI ≥ 3 (≥4 vaccinations: aHR = 0.29, 95% CI: 0.18, 0.46), and dyslipidemia (≥4 vaccinations: aHR = 0.30, 95% CI: 0.19, 0.48). The results of the sensitivity analysis of the aHRs in the noninfluenza season are listed in Table 4. A stronger protective effect against ischemic stroke was observed during the noninfluenza season. Less frequent vaccination significantly reduced the risk of ischemic stroke. Vaccination at a frequency of 2 or 3 times conferred a protective effect on the patients with AF. The protective effect was more predominant in the patients aged ≥75 y (1 vaccination: aHR = 0.64, 95% CI: 0.44, 0.94; 2 or 3 vaccinations: aHR = 0.56, 95% CI: 0.39, 0.81; and ≥4 vaccinations: aHR = 0.23, 95% CI: 0.14, 0.39) and in those with diabetes (2 or 3 vaccinations: aHR = 0.35, 95% CI: 0.20, 0.61; ≥4 vaccinations: aHR = 0.20, 95% CI: 0.10, 0.38), dyslipidemia (2 or 3 vaccinations: aHR = 0.38, 95% CI: 0.22, 0.68; ≥4 vaccinations: aHR = 0.19, 95% CI: 0.10, 0.39), hypertension (2 or 3 vaccinations: aHR = 0.57, 95% CI: 0.41, 0.78; ≥4 vaccinations: aHR = 0.25, 95% CI: 0.17, 0.38), and CCI ≥ 3 (2 or 3 vaccinations: aHR = 0.40, 95% CI: 0.34, 0.67; ≥4 vaccinations: aHR = 0.25, 95% CI: 0.14, 0.46). During all seasons (Table 5), the trend of ischemic stroke reduction still reflected the vaccination frequency. The protective effect was more predominant in the patients aged ≥75 y (1 vaccination: aHR = 0.68, 95% CI: 0.54, 0.87; 2 or 3 vaccinations: aHR = 0.49, 95% CI: 0.38, 0.64; and ≥4 vaccinations: aHR = 0.30, 95% CI: 0.23, 0.41) and in those with diabetes (2 or 3 vaccinations: aHR = 0.52, 95% CI: 0.38, 0.72; ≥4 vaccinations: aHR = 0.29, 95% CI: 0.20, 0.43), dyslipidemia (2 or 3 vaccinations: aHR = 0.61, 95% CI: 0.45, 0.84; ≥4 vaccinations: aHR = 0.26, 95% CI: 0.17, 0.37), hypertension (2 or 3 vaccinations: aHR = 0.60, 95% CI: 0.49, 0.73; ≥4 vaccinations: aHR = 0.33, 95% CI: 0.26, 0.42), and CCI ≥ 3 (2 or 3 vaccinations: aHR = 0.44, 95% CI: 0.32, 0.60; ≥4 vaccinations: aHR = 0.27, 95% CI: 0.19, 0.39). Regardless of warfarin, statin, metformin, ACEI, or aspirin use, vaccination was an independent protective factor and dose-dependently reduced the risk of ischemic stroke in the patients with AF.

**Table 3:** Sensitivity analysis of aHRs of vaccination in risk reduction of ischemic stroke in the influenza season

	Unvaccinated patients	Vaccinated patients			*P* for trend
		1 vaccination	2 or 3 vaccinations	≥ 4 vaccinations	
	aHR (95% CI)	aHR (95% CI)	aHR (95% CI)	aHR (95% CI)	
Main model^†^	1.00	0.91 (0.72, 1.15)	0.62 (0.49, 0.79)^***^	0.40 (0.31, 0.51)^***^	<0.001
**Additional covariates^‡^**					
Main model + warfarin	1.00	0.90 (0.71, 1.14)	0.62 (0.49, 0.79)^***^	0.39 (0.30, 0.50)^***^	<0.001
Main model + aspirin	1.00	0.91 (0.72, 1.14)	0.62 (0.49, 0.79)^***^	0.40 (0.31, 0.51)^***^	<0.001
Main model + statin	1.00	0.91 (0.72, 1.15)	0.62 (0.49, 0.79)^***^	0.40 (0.31, 0.51)^***^	<0.001
Main model + ACEI	1.00	0.91 (0.72, 1.15)	0.62 (0.49, 0.79)^***^	0.40 (0.31, 0.51)^***^	<0.001
Main model + metformin	1.00	0.91 (0.72, 1.14)	0.62 (0.49, 0.78)^***^	0.39 (0.31, 0.51)^***^	<0.001
Subgroup effects					
Age, y					
55–74	1.00	1.09 (0.77, 1.53)	0.87 (0.63, 1.21)	0.50 (0.36, 0.70)^***^	<0.001
≥75	1.00	0.71 (0.52, 0.97)^*^	0.45 (0.31, 0.63)^***^	0.35 (0.24, 0.51)^***^	<0.001
Sex					
Women	1.00	0.65 (0.45, 0.94)^*^	0.47 (0.33, 0.68)^***^	0.41 (0.29, 0.58)^***^	<0.001
Men	1.00	1.22 (0.90, 1.67)	0.81 (0.59, 1.13)	0.40 (0.28, 0.57)^***^	<0.001
CCI^+^					
0	1.00	0.62 (0.34, 1.13)	0.57 (0.32, 1.00)	0.39 (0.22, 0.68)^***^	0.001
1	1.00	0.81 (0.47, 1.37)	0.74 (0.46, 1.18)	0.46 (0.28, 0.74)^**^	0.002
2	1.00	1.21 (0.76, 1.91)	0.64 (0.38, 1.07)	0.40 (0.24, 0.66)^***^	<0.001
≥3	1.00	0.87 (0.60, 1.26)	0.46 (0.31, 0.70)^***^	0.29 (0.18, 0.46)^***^	<.001
Diabetes					
No	1.00	0.86 (0.64, 1.15)	0.57 (0.43, 0.78)^***^	0.39 (0.29, 0.53)^***^	<0.001
Yes	1.00	0.97 (0.66, 1.42)	0.66 (0.45, 0.99)^***^	0.37 (0.23, 0.59)^***^	<0.001
Dyslipidemia					
No	1.00	1.00 (0.76, 1.32)	0.52 (0.38, 0.72)^***^	0.42 (0.31, 0.57)^***^	<0.001
Yes	1.00	0.71 (0.46, 1.08)	0.78 (0.54, 1.13)	0.30 (0.19, 0.48)^***^	<0.001
Hypertension					
No	1.00	0.53 (0.31, 0.92)^*^	0.57 (0.34, 0.94)^*^	0.36 (0.22, 0.60)^***^	<0.001
Yes	1.00	1.04 (0.80, 1.35)	0.62 (0.47, 0.81)^***^	0.39 (0.29, 0.53)^***^	<0.001
Warfarin					
<28 cDDD	1.00	0.93 (0.69, 1.24)	0.56 (0.41, 0.76)^***^	0.39 (0.28, 0.54)^***^	<0.001
≥28 cDDD	1.00	0.85 (0.58, 1.26)	0.71 (0.49, 1.03)	0.39 (0.26, 0.56)^***^	<0.001
Aspirin					
<28 cDDD	1.00	0.90 (0.55, 1.47)	0.49 (0.28, 0.85)^**^	0.42 (0.22, 0.80)^**^	<0.001
≥28 cDDD	1.00	0.87 (0.67, 1.14)	0.64 (0.49, 0.84)^**^	0.37 (0.28, 0.49)^***^	<0.001
Statin					
<28 cDDD	1.00	0.89 (0.67, 1.18)	0.53 (0.39, 0.72)^***^	0.43 (0.32, 0.59)^***^	<0.001
≥28 cDDD	1.00	0.93 (0.62, 1.41)	0.76 (0.52, 1.11)	0.34 (0.22, 0.51)^***^	<0.001
ACEI					
<28 cDDD	1.00	0.58 (0.34, 1.02)	0.49 (0.28, 0.85)^*^	0.33 (0.17, 0.62)^***^	<0.001
≥28 cDDD	1.00	1.01 (0.78, 1.32)	0.67 (0.51, 0.87)^**^	0.42 (0.32, 0.55)^***^	<0.001
Metformin					
<28 cDDD	1.00	0.83 (0.63, 1.10)	0.59 (0.44, 0.78)^***^	0.41 (0.31, 0.54)^***^	<0.001
≥28 cDDD	1.00	1.09 (0.70, 1.70)	0.68 (0.43, 1.08)	0.35 (0.21, 0.58)^***^	<0.001

^*^P < 0.05 ^**^P < 0.01 ^***^P < 0.001

HR: hazard ratio

aHR: adjusted hazard ratio

^+^CCI: Charlson comorbidity index

^†^The main model was adjusted for age; sex; Charlson comorbidity index; comorbidities of diabetes, hypertension, dyslipidemia, congestive heart failure, vascular disease, pneumonia, and dialysis; urbanization level; and monthly income by using propensity scores.

^‡^The models were adjusted for covariates in the main model and each additional listed covariate.

**Table 4:** Sensitivity analysis of aHRs of vaccination in risk reduction of ischemic stroke in the noninfluenza season

	Unvaccinated patients	Vaccinated patients			*P* for trend
		1	2 or 3	≥ 4	
	aHR (95% CI)	aHR (95% CI)	aHR (95% CI)	aHR (95% CI)	
**Main model^†^**	1.00	0.73 (0.55, 0.97)^*^	0.58 (0.44, 0.77)^***^	0.28 (0.20, 0.40)^***^	<0.001
**Additional covariates^‡^**					
Main model + warfarin	1.00	0.73 (0.55, 0.98)^*^	0.58 (0.44, 0.77)^***^	0.29 (0.21, 0.40)^***^	<0.001
Main model + aspirin	1.00	0.74 (0.55, 0.98)^*^	0.60 (0.45, 0.79)^***^	0.29 (0.21, 0.41)^***^	<0.001
Main model + statin	1.00	0.73 (0.55, 0.97)^*^	0.59 (0.44, 0.78)^***^	0.28 (0.20, 0.40)^***^	<0.001
Main model + ACEI	1.00	0.73 (0.55, 0.98)^*^	0.59 (0.44, 0.78)^***^	0.28 (0.20, 0.40)^***^	<0.001
Main model + metformin	1.00	0.73 (0.55, 0.97)^*^	0.58 (0.44, 0.77)^***^	0.28 (0.20, 0.39)^***^	<0.001
Subgroup effects					
Age, y					
55–74	1.00	0.78 (0.51, 1.19)	0.62 (0.41, 0.94)^*^	0.38 (0.25, 0.59)^***^	<0.001
≥75	1.00	0.64 (0.44, 0.94)^*^	0.56 (0.39, 0.81)^**^	0.23 (0.14, 0.39)^***^	<0.001
Sex					
Women	1.00	0.83 (0.56, 1.23)	0.62 (0.42, 0.92)^*^	0.27 (0.16, 0.44)^***^	<0.001
Men	1.00	0.64 (0.43, 0.97)^*^	0.55 (0.37, 0.81)^**^	0.29 (0.18, 0.45)^***^	<0.001
CCI^+^					
0	1.00	0.91 (0.47, 1.74)	0.76 (0.39, 1.48)	0.49 (0.25, 0.96)^*^	0.037
1	1.00	0.51 (0.25, 1.04)	0.74 (0.43, 1.26)	0.33 (0.18, 0.62)^***^	0.001
2	1.00	0.67 (0.37, 1.23)	0.55 (0.30, 0.99)^*^	0.29 (0.19, 0.48)^***^	<0.001
≥3	1.00	0.76 (0.49, 1.20)	0.40 (0.24, 0.67)^***^	0.25 (0.14, 0.46)^***^	<0.001
Diabetes					
No	1.00	0.64 (0.44, 0.94)^*^	0.72 (0.52, 1.00)	0.33 (0.22, 0.49)^***^	<0.001
Yes	1.00	0.86 (0.56, 1.33)	0.35 (0.20, 0.61)^***^	0.20 (0.10, 0.38)^***^	<0.001
Dyslipidemia					
No	1.00	0.56 (0.38, 0.83)^**^	0.68 (0.49, 0.94)^*^	0.33 (0.23, 0.49)^***^	<0.001
Yes	1.00	1.05 (0.68, 1.61)	0.38 (0.22, 0.68)^***^	0.19 (0.10, 0.36)^***^	<0.001
Hypertension					
No	1.00	0.69 (0.38, 1.25)	0.56 (0.30, 1.04)	0.36 (0.19, 0.68)^**^	<0.001
Yes	1.00	0.74 (0.54, 1.03)	0.57 (0.41, 0.78)^***^	0.25 (0.17, 0.38)^***^	<0.001
Warfarin					
<28 cDDD	1.00	0.82 (0.59, 1.12)	0.56 (0.40, 0.78)^***^	0.26 (0.17, 0.40)^***^	<0.001
≥28 cDDD	1.00	0.51 (0.27, 0.96)^*^	0.65 (0.38, 1.11)	0.32 (0.18, 0.57)^***^	<0.001
Aspirin					
<28 cDDD	1.00	0.69 (0.35, 1.37)	0.58 (0.31, 1.10)	0.44 (0.21, 0.92)^*^	0.011
≥28 cDDD	1.00	0.71 (0.51, 0.97)^*^	0.57 (0.42, 0.78)^***^	0.25 (0.17, 0.36)^***^	<0.001
Statin					
<28 cDDD	1.00	0.64 (0.45, 0.92)^*^	0.50 (0.35, 0.71)^***^	0.29 (0.19, 0.44)^***^	<0.001
≥28 cDDD	1.00	0.93 (0.57, 1.53)	0.75 (0.47, 1.19)	0.27 (0.15, 0.48)^***^	<0.001
ACEI					
<28 cDDD	1.00	0.37 (0.17, 0.80)^*^	0.35 (0.17, 0.73)^**^	0.47 (0.24, 0.91)^*^	<0.001
≥28 cDDD	1.00	0.86 (0.63, 1.17)	0.66 (0.48, 0.90)^**^	0.26 (0.18, 0.38)^***^	<0.001
Metformin					
<28 cDDD	1.00	0.66 (0.46, 0.93)^*^	0.64 (0.46, 0.88)^**^	0.30 (0.20, 0.44)^***^	<0.001
≥28 cDDD	1.00	0.93 (0.56, 1.55)	0.45 (0.25, 0.82)^**^	0.25 (0.13, 0.49)^***^	<0.001

^*^P < 0.05 ^**^P < 0.01 ^***^P < 0.001

HR: hazard ratio

aHR: adjusted hazard ratio

^+^CCI: Charlson comorbidity index

^†^The main model was adjusted for age; sex; Charlson comorbidity index; comorbidities of diabetes, hypertension, dyslipidemia, congestive heart failure, vascular disease, pneumonia, and dialysis; urbanization level; and monthly income by using propensity scores.

^‡^The models were adjusted for covariates in the main model and each additional listed covariate.

**Table 5:** Sensitivity analysis of aHRs of vaccination in risk reduction of ischemic stroke in all seasons

	Unvaccinated patients	Vaccinated patients			*P* for trend
		1	2 or 3	≥ 4	
	aHR (95% CI)	aHR (95% CI)	aHR (95% CI)	aHR (95% CI)	
**Main model^†^**	1.00	0.83 (0.69, 1.00)^*^	0.60 (0.50, 0.72)^***^	0.35 (0.29, 0.43)^***^	<0.001
**Additional covariates^‡^**					
Main model + warfarin	1.00	0.83 (0.69, 0.99)^*^	0.60 (0.50, 0.73)^***^	0.34 (0.28, 0.42)^***^	<0.001
Main model + aspirin	1.00	0.83 (0.69, 1.00)^*^	0.61 (0.51, 0.73)^***^	0.35 (0.29, 0.43)^***^	<0.001
Main model + statin	1.00	0.83 (0.69, 1.00)^*^	0.60 (0.50, 0.73)^***^	0.35 (0.29, 0.43)^***^	<0.001
Main model + ACEI	1.00	0.83 (0.69, 1.00)^*^	0.60 (0.50, 0.73)^***^	0.35 (0.29, 0.43)^***^	<0.001
Main model + metformin	1.00	0.83 (0.69, 0.99)^*^	0.60 (0.50, 0.72)^***^	0.35 (0.28, 0.42)^***^	<0.001
Subgroup effects					
Age, y					
55–74	1.00	0.95 (0.73, 1.24)	0.76 (0.59, 0.98)^*^	0.45 (0.35, 0.59)^***^	<0.001
≥75	1.00	0.68 (0.54, 0.87)^**^	0.49 (0.38, 0.64)^***^	0.30 (0.23, 0.41)^***^	<0.001
Sex					
Women	1.00	0.72 (0.55, 0.94)^*^	0.53 (0.41, 0.69)^***^	0.35 (0.27, 0.47)^***^	<0.001
Men	1.00	0.95 (0.74, 1.21)	0.69 (0.53, 0.88)^**^	0.35 (0.26, 0.46)^***^	<0.001
CCI^+^					
0	1.00	0.73 (0.47, 1.14)	0.64 (0.42, 0.99)^*^	0.43 (0.28, 0.66)^***^	<0.001
1	1.00	0.67 (0.44, 1.03)	0.74 (0.52, 1.05)	0.40 (0.28, 0.59)^***^	<0.001
2	1.00	0.95 (0.66, 1.37)	0.59 (0.40, 0.88)^**^	0.27 (0.18, 0.42)^***^	<0.001
≥3	1.00	0.83 (0.62, 1.10)	0.44 (0.32, 0.60)^***^	0.27 (0.19, 0.39)^***^	<0.001
Diabetes					
No	1.00	0.77 (0.61, 0.97)^*^	0.63 (0.51, 0.79)^***^	0.37 (0.29, 0.47)^***^	<0.001
Yes	1.00	0.92 (0.69, 1.23)	0.52 (0.38, 0.72)^***^	0.29 (0.20, 0.43)^***^	<0.001
Dyslipidemia					
No	1.00	0.81 (0.64, 1.01)	0.59 (0.47, 0.74)^***^	0.38 (0.30, 0.49)^***^	<0.001
Yes	1.00	0.85 (0.63, 1.15)	0.61 (0.45, 0.84)^**^	0.26 (0.17, 0.37)^***^	<0.001
Hypertension					
No	1.00	0.59 (0.40, 0.89)^*^	0.56 (0.38, 0.83)^***^	0.36 (0.24, 0.54)^***^	<0.001
Yes	1.00	0.91 (0.74, 1.11)	0.60 (0.49, 0.73)^***^	0.33 (0.26, 0.42)^***^	<0.001
Warfarin					
<28 cDDD	1.00	0.87 (0.70, 1.08)	0.56 (0.44, 0.70)^***^	0.33 (0.25, 0.43)^***^	<0.001
≥28 cDDD	1.00	0.73 (0.52, 1.01)	0.69 (0.51, 0.93)^*^	0.36 (0.27, 0.50)^***^	<0.001
Aspirin					
<28 cDDD	1.00	0.82 (0.55, 1.22)	0.52 (0.34, 0.80)^**^	0.43 (0.26, 0.70)^***^	<0.001
≥28 cDDD	1.00	0.80 (0.65, 0.98)^*^	0.61 (0.50, 0.74)^***^	0.32 (0.26, 0.40)^***^	<0.001
Statin					
<28 cDDD	1.00	0.78 (0.63, 0.97)^*^	0.51 (0.41, 0.65)^***^	0.37 (0.29, 0.47)^***^	<0.001
≥28 cDDD	1.00	0.93 (0.68, 1.28)	0.76 (0.56, 1.01)	0.31 (0.22, 0.44)^***^	<0.001
ACEI					
<28 cDDD	1.00	0.49 (0.31, 0.77)^**^	0.43 (0.28, 0.67)^***^	0.38 (0.24, 0.61)^***^	<0.001
≥28 cDDD	1.00	0.95 (0.77, 1.16)	0.66 (0.54, 0.81)^***^	0.35 (0.28, 0.44)^***^	<0.001
Metformin					
<28 cDDD	1.00	0.76 (0.61, 0.94)^*^	0.61 (0.49, 0.75)^***^	0.36 (0.29, 0.46)^***^	<0.001
≥28 cDDD	1.00	1.02 (0.73, 1.42)	0.58 (0.40, 0.83)^***^	0.31 (0.21, 0.46)^***^	<0.001

^*^P < 0.05 ^**^P < 0.01 ^***^P < 0.001

HR: hazard ratio

aHR: adjusted hazard ratio

^+^CCI: Charlson comorbidity index

^†^The main model was adjusted for age; sex; Charlson comorbidity index; comorbidities of diabetes, hypertension, dyslipidemia, congestive heart failure, vascular disease, pneumonia, and dialysis; urbanization level; and monthly income by using propensity scores.

^‡^The models were adjusted for covariates in the main model and each additional listed covariate.

## DISCUSSION

To date, few studies have investigated the association of the risk of ischemic stroke with influenza vaccination in patients with AF who received influenza vaccination. Hung et al. conducted a cohort study and reported that the efficacy of the dual influenza and pneumococcal vaccine is higher than that of either vaccine alone in preventing complications in elderly patients with chronic illnesses [19]. Moreover, the rate of ischemic stroke (HR = 0.67; 95% CI: 0.54, 0.83) was lower in elderly patients who were aged ≥65 y, had chronic illnesses, and received the dual influenza and pneumococcal polysaccharide vaccine than in unvaccinated patients [19]. The population and intervention used in our study differ from those used in the study of Hung et al. In our study, the intervention was influenza vaccination exhibiting a more specific efficacy than that used by Hung et al [19]. Although studies have used a similar population of elderly patients,[13, 14] they did not focus on patients having a high risk of ischemic stroke (i.e., patients with AF). In this study, we included a specific AF population with a risk of ischemic stroke. Our study results reveal that influenza vaccination was an independent protective factor and dose-dependently reduced the risk of ischemic stroke in the patients with AF, irrespective of age, sex, hypertension, heart disease, or anticoagulant use. According to our review of the literature, this is the first study to provide a valuable solution for reducing the risk of ischemic stroke in patients with AF. This strategy for ischemic stroke prevention in patients with AF requires further investigation.

The precise mechanism through which influenza vaccination reduces the risk of ischemic stroke in patients with AF remains unknown. The protective effect of influenza vaccination is likely related to the prevention of acute infection, which can elicit both systemic and local vascular inflammatory responses [20]. Influenza increases local proinflammatory cytokine expression, platelet aggregation, and systemic inflammation marker levels and causes endothelial dysfunction and loss of the protective properties of high-density lipoproteins [21–24]. All these changes can directly or indirectly stimulate thrombogenesis and exacerbate atherosclerotic plaque inflammation, which can increase the risk of stroke in combination with increased hemodynamic stress. In addition, in an experimental human influenza A study, patients treated with an oral neuraminidase inhibitor had lower levels of the proinflammatory cytokines interferon-γ, interleukin-6, and tumor necrosis factor-α in nasal washings than did those treated with a placebo. In the placebo group, the levels of these cytokines were 2- to 4-fold higher than baseline levels [25]. Considering the potential proinflammatory and prothrombotic consequences of influenza, influenza vaccination might prevent the high expression of proinflammatory cytokines and reduce inflammation, viral load, and illness duration, thereby exerting a positive effect on the risk of thrombotic events such as stroke induced by influenza infection. During and after influenza epidemics, vascular death rates and hospitalizations for stroke increased [26]. Studies have reported that influenza might be the cause of this acute infection, leading to ischemic stroke,[19, 27] particularly in patients with AF who are susceptible to ischemic stroke. The Taiwan Centers for Disease Control included >70% of circulating influenza strains in the influenza vaccine [28] and successfully reduced the rate of influenza transmission[29, 30] and risk of AF through influenza vaccination [17]. In addition, if a mismatch occurs between the circulating influenza strains and vaccine antigens, the effect of the influenza vaccine would be underestimated, thus leading to “bias toward the null hypothesis.” However, the actual effect of the influenza vaccine would be high if the circulating influenza strains and vaccine antigens match. In our study, we observed that influenza vaccination independently exerted a strong dose-dependent effect on ischemic stroke prevention in the patients with AF.

The unvaccinated and vaccinated patients had different baseline characteristics (Table 1). In Taiwan, influenza vaccination has been provided free of charge and recommended for high-risk adults aged ≥55 y (i.e., those with hypertension, congestive heart failure, chronic liver infection, liver cirrhosis, cardiovascular diseases, or chronic pulmonary diseases) since 1998 and for all adults aged >65 y since 2001. We selected covariates on the basis of a logistic regression model. Each patient was followed to assess the risk of and protective factors for ischemic stroke. We used PSs to evaluate the following demographic characteristics: age; sex; CCI; comorbidities of diabetes, hypertension, dyslipidemia, congestive heart failure, vascular disease, pneumonia, and dialysis; urbanization level; monthly income; and warfarin, statin, metformin, aspirin, and ACEI use (Supplementary Table 1).

In patients with AF, a cardiac embolus most commonly originating from the left atrium is a common cause of ischemic stroke [31]. A cardioembolic event is likely to have occurred in patients with AF who have had a stroke [31, 32]. AF is common in elderly patients who often have a risk of other stroke types [6]. Thus, the presence of AF in a patient who had a stroke does not always imply a causal relationship [6]. Consequently, all patients with stroke, even in the presence of AF, should be investigated for other causes of stroke, particularly if such an investigation would result in different treatments. On the basis of this rationale, we investigated whether influenza vaccination reduces the risk of hemorrhagic stroke in patients with AF (data not published). The CHA2DS2-VASc score is a clinical prediction rule used to estimate the risk of stroke in patients with AF [33, 34]. Risk factors included in the CHA2DS2-VASc score are congestive heart failure, hypertension, age (65–74 or ≥75 y), diabetes mellitus, prior stroke, vascular disease, or female sex [33, 34]. Hypertension is a crucial component in the management of patients with AF who have had a stroke [35]. Antihypertensive therapy, preferably including an ACEI,[36] reduces the risk of warfarin-associated intracranial hemorrhage and may reduce the rate of recurrent stroke [37]. Hence, we selected these risk factors as covariates in the main model or as an additional covariate in the sensitivity analysis (Tables 2–5).

The risk factors in the CHA2DS2-VASc score included being aged ≥75 y (2 points) and 65–74 y (1 point) [33, 34]. Our data reveal that age is a strong risk factor for ischemic stroke in the patients with AF. The risk of ischemic stroke was higher in the elderly patients than in the young patients. Table 2 presents the risk of ischemic stroke in the unvaccinated and vaccinated patients. The stratified analysis after adjustment for PSs in the main model demonstrated that aHRs significantly decreased in the vaccinated patients, particularly in those aged 65–74 and ≥75 y. The aHRs of ischemic stroke were lower in the vaccinated patients than in the unvaccinated patients during all seasons. Notably, despite the small sample size of the 55–64-y-old age group, the aHRs remained significantly lower in the vaccinated patients during the noninfluenza season. A competing risk factor might be present between the influenza and noninfluenza seasons in the 55–64-y-old age group [20]. In this study, influenza vaccination exerted a stronger protective effect in more elderly patients (65–74 and ≥75 y) with AF. This finding indicates the importance of administering influenza vaccination to elderly patients and elderly patients with AF having a relatively high risk of ischemic stroke. In the 55–64-y-old age group, influenza vaccination exerted a protective effect during the noninfluenza season. This finding indicates that influenza vaccination is crucial for working-age (<65 y) patients with AF.

Regarding the female sex, a retrospective cohort study of approximately 100 000 patients with AF conducted in 2012 reported that the risk of ischemic stroke was moderately higher in women [38]. The female sex is included as a risk factor in the CHA2DS2-VASc score [33, 34]. Our study results reveal that the female sex was associated with a high risk of ischemic stroke in the patients with AF. The stratified analysis after adjustment for PSs in the main model revealed that the aHRs were significantly lower in the vaccinated patients, irrespective of sex, age, or season. The aHRs of ischemic stroke were lower in the vaccinated patients than in the unvaccinated patients during all seasons (women and men: aHRs = 0.51 and 0.61; *P* < 0.001 and *P* < 0.001, respectively). In this study, influenza vaccination conferred a stronger protective effect on the female patients with AF. Biological and physiological characteristics defining women and men may exert differential effects on disease outcomes after pathogen exposure. In addition, women and men differ in their reproductive organs and thus sex hormone concentrations. Studies conducted in Denmark and Canada using hospitalization rates as an indicator of influenza severity have reported that the risk of influenza was higher in men than in women across all age groups during influenza virus outbreaks [39, 40]. A study conducted in Switzerland demonstrated an increased influenza-related death rate in men (aged >60 y) during the 1969–1999 season,[41] partly supporting the findings from Denmark and Canada. Overall, men had a higher risk of influenza-related mortality, with a male-to-female ratio of 1:3 [42]. A competing risk factor may be present between men and women during influenza and noninfluenza seasons, which may be the reason for our observation of significantly lower aHRs in the vaccinated patients during the influenza season, particularly in the female patients. By contrast, the aHRs were significantly lower in the vaccinated male patients during the noninfluenza season (Table 2).

As presented in Tables 3 and 4, influenza vaccination dose-dependently reduced the risk of ischemic stroke in all the subgroups and the main model with additional covariates (warfarin, statin, metformin, ACEI, or aspirin use) during different seasons. According to all the aHRs, vaccination significantly reduced the risk of ischemic stroke in all the subgroups, regardless of comorbidities or drug use (*P* < 0.001). The outcomes for vaccination imply that this intervention may exert an independent protective effect against the risk of ischemic stroke in patients with AF. No study has evaluated the protective effect of influenza vaccination in patients with drug use, which might reduce the risk of stroke in patients with AF. This is the first study to investigate the effect of influenza vaccination on the risk of ischemic stroke in patients with AF with or without drug use. Our study results reveal that influenza vaccination independently exerted a dose-response effect against ischemic stroke in the patients with AF, regardless of the presence of diabetes, hypertension, dyslipidemia, congestive heart failure, or vascular disease or statin, metformin, warfarin, or ACEI use.

We obtained several major novel findings. Few studies have investigated the association of influenza vaccination with the risk of ischemic stroke in patients with AF. Although studies have reported that the risk of influenza-related mortality and ischemic stroke is high in patients with AF, adequate evidence on solutions to reduce the mortality and risk of ischemic stroke in patients with AF is not available. Our findings indicate that influenza vaccination exerted a dose-dependent protective effect against the risk of ischemic stroke in the patients with AF. The major solution might be the regular administration of influenza vaccine to patients with AF, particularly to those having risk factors for ischemic stroke, such as hypertension, dyslipidemia, diabetes, cerebrovascular disease, and a high CHADS2 score. Tables 3–5 list the results of the sensitivity analysis of the aHRs of age, sex, CCI, comorbidities, urbanization level, and monthly income in the PS analysis. The models were adjusted for covariates in the main model and each additional covariate to estimate the reduction in the risk of ischemic stroke during the follow-up period. The dose-dependent protective effect of influenza vaccination was observed regardless of age, sex, CCI, comorbidities, urbanization level, monthly income, or drug use in the analysis stratified by different frequencies of influenza vaccination. The dose-dependent protective effect of influenza vaccination was observed for different conditional states. In addition, this study is the first to evaluate the dose-response effect of influenza vaccination on the risk of ischemic stroke in patients with AF. Our results reveal that only one influenza vaccination was less effective in reducing the risk of ischemic stroke. A high frequency of influenza vaccination exerted a more significant protective effect against ischemic stroke in the patients with AF. The strength of the present study is its large sample size. The results suggest that the incidence of ischemic stroke decreased in the patients with AF through the implementation of preventive strategies such as influenza vaccination. This is the first study to demonstrate that influenza vaccination exerts a dose-response effect against ischemic stroke in patients with AF who have risk factors for ischemic stroke by reducing the incidence of ischemic stroke, particularly in patients aged 65–74 and ≥75 y.

In this study, the magnitude of the bias demonstrated by the associations observed during the noninfluenza season was sufficient to entirely account for the associations observed during the influenza season. A competing risk factor is present between the influenza and noninfluenza seasons. Studies have reported an increased risk of hospital admission and a high mortality rate among elderly men during the influenza season [43–45]. High mortality during the influenza season might mask the incidence of ischemic stroke episodes, and patients with AF might die before developing ischemic stroke. This observation is attributable to influenza vaccination exerting a stronger protective effect against ischemic stroke during the noninfluenza season (Tables 3 and 4). We noted a similar phenomenon in our previous study [20].

In Taiwan, the most frequently used vaccines are influenza and pneumococcal vaccines. However, introduction of the pneumococcal vaccine into the national immunization program is complex and costly. With financial support from a nongovernmental organization, the pneumococcal vaccine has been provided to elderly people aged ≥75 y since 2007 [46]. The overall vaccination rate was <1% in Taiwan before 2007 [47]. Compared with the influenza vaccine, the administration of other vaccines was relatively rare. These rare cases might not influence our results. Moreover, considering the magnitude and significance of the observed effects, it is unlikely that these limitations compromised the results.

This study has some potential limitations. Observational studies have suggested that lifestyle factors, particularly ethnicity, family history, genetic disorders, physical activity, and other potential confounding factors, are associated with the risk of ischemic stroke. However, methodological concerns may obscure the precise relationship between these factors and the risk of ischemic stroke. In this study, we used PSs to adjust age, sex, CCI, comorbidities, urbanization level, and monthly income. The urbanization level and monthly income are invalidated alternatives for lifestyle factors. To obtain such information, a large randomized trial applying a suitable regimen to well-selected patients to compare standard approaches is necessary. Moreover, the diagnoses of ischemic stroke and all other comorbidities were completely dependent on ICD-9-CM codes. However, the National Health Insurance Administration randomly reviews charts and interviews patients to validate diagnoses. A study conducted in Taiwan reported a positive predictive value (PPV) of 88.4% (95% CI: 86.8%, 89.8%) and sensitivity of 97.3% (95% CI, 96.4%, 98.1%) for diagnoses. The PPV of the diagnosis of ischemic stroke, AF, or a disease included in the NHI claims data was high [48, 49]. Hospitals with outlier diagnoses and practices may be audited and subsequently heavily penalized if malpractices or discrepancies are discovered. In the absence of actual patient exposure to influenza disease (as evidenced by antibody titers), deriving a mechanism for administration of the vaccine to reduce the risk of ischemic stroke will be challenging. A randomized controlled trial should be designed to test the viral serotype frequency in a cohort to verify actual patient exposure to influenza disease. However, our study results indicate a possibility that a high frequency of influenza vaccination exerted a significant protective effect against ischemic stroke in the patients with AF. Another limitation is that several unmeasured confounders, including body mass index, smoking, alcohol intake, and over-the-counter drug use, associated with ischemic stroke are not available in the NHIRD. However, considering the magnitude and significance of the observed effects, it is unlikely that these limitations compromised the results. Finally, our study was not a prospective randomized blinded study; thus, a cause–effect relationship could not be established. The findings of the present study suggest that influenza vaccination exerts a significant protective effect. Randomized studies are required to verify these findings.

## MATERIALS AND METHODS

The National Health Insurance (NHI) program, established in 1995, currently provides comprehensive health insurance coverage to 98% of more than 23 million people in Taiwan. In this study, we used data from the National Health Insurance Research Database (NHIRD). No significant differences have been observed in age, sex, or health care costs between people sampled in the NHIRD and all NHI enrollees. According to the Taiwan Centers for Disease Control, the influenza season extends from October to March. Data in the NHIRD that can be used to identify patients or care providers, including medical institutions and physicians, are encrypted before being sent to the National Health Research Institutes for database construction and are further scrambled before being released to researchers. Theoretically, querying the data alone to identify people at any level is impractical. All researchers using the NHIRD and its data subsets must sign a written agreement declaring that they have no intention of attempting to obtain information that could potentially violate the privacy of patients or care providers [18, 19].

The study cohort comprised all patients diagnosed as having AF (according to International Classification of Diseases, Ninth Revision, Clinical Modification [ICD-9-CM] codes) at health care facilities in Taiwan (n = 14 454) before January 1, 2005. The last follow-up date was December 31, 2013. We excluded all patients without a subsequent outpatient visit, emergency department visit, or inpatient hospitalization for AF within 12 mo of the first presentation (n = 1825) because they were considered to not have AF. In addition, we excluded 6059 patients who were younger than 55 y (n = 1699), had any inpatient or outpatient diagnosis related to stroke before the enrollment date, did not die of ischemic stroke before December 31, 2013 (n = 2236), or had received an influenza or pneumococcal polysaccharide vaccine before the enrollment date (n = 2124).

In Taiwan, influenza vaccination has been provided free of charge and recommended for high-risk adults aged ≥55 y (i.e., those with type 2 diabetes, chronic liver infection, liver cirrhosis, cardiovascular diseases, or chronic pulmonary diseases) since 1998 and for all adults aged >65 y since 2001. In this study, the vaccination status was identified using the ICD-9-CM code V048 and/or identified on the basis of vaccine use (confirmed using drug codes) [19, 20]. Our final study cohort comprised 6570 patients diagnosed as having AF in Taiwan before January 1, 2005. Of these patients, 2547 and 4023 received and did not receive influenza vaccination, respectively. We selected covariates on the basis of a logistic regression model. Each patient was followed to assess the risk of and protective factors for ischemic stroke. We used propensity scores (PSs) to evaluate the following demographic characteristics: age; sex; Charlson comorbidity index (CCI); comorbidities of diabetes, hypertension, dyslipidemia, congestive heart failure, vascular disease, pneumonia, and dialysis; urbanization level; monthly income; and warfarin, statin, metformin, aspirin, and angiotensin-converting enzyme inhibitor (ACEI) use (Supplementary Table 1). All potential confounders (Supplementary Table 1) were included in the list of regressors (C statistic: 0.68). Moreover, all potential confounders observed within 6 mo before and after the index date until the endpoint (ischemic stroke) were identified according to the main diagnosis code for the first admission or according to more than 2 repeated main diagnosis codes for visits to an outpatient department. The patients who received the prescribed drugs for <28 cumulative defined daily doses (cDDDs) were defined as nonusers. We derived PSs by using a logistic regression model to estimate the effect of vaccination by accounting for covariates that predicted receiving the intervention (vaccine). This method was used in an observational study to reduce selection bias [21]. The covariates in the main model were adjusted for the PSs of age, sex, CCI, comorbidities, urbanization level (urban, suburban, and rural), and monthly income (none; NT$1–NT$21,000; NT$21,000–NT$33,300; and ≥NT$33,301; NT$ represents New Taiwan dollars; Table 2). The endpoint was the incidence of ischemic stroke (ICD-9-CM codes 433–437) in the vaccinated or unvaccinated patients with a subsequent outpatient visit, emergency department visit, or inpatient hospitalization for ischemic stroke within 12 mo; moreover, the unvaccinated patients served as the reference arm. Because the protective effect of each vaccination is specific to that influenza season, evaluating the noninfluenza season can indicate the possible contribution of bias to estimates obtained during the influenza season. In addition, the relationship between the seasonal effect of vaccination and the risk of ischemic stroke was analyzed. The cumulative incidence of ischemic stroke in the vaccinated and unvaccinated patients with AF was estimated using the Kaplan–Meier method. To examine the effect of the total number of vaccinations on the cumulative incidence of ischemic stroke, we categorized the patients into 4 groups according to the vaccination status: unvaccinated and 1, 2 to 3, and ≥4 total vaccinations.

We used a Cox proportional hazard model with time-dependent covariates to prevent immortal time bias. A Cox proportional hazard model was used to calculate the hazard ratios (HRs) of ischemic stroke in the vaccinated and unvaccinated patients with AF [22]. The index date was January 1, 2005. All covariates observed 6 mo before and after the index date were included. In a multivariate analysis, the HRs were adjusted for age; sex; CCI; comorbidities of diabetes, hypertension, dyslipidemia, congestive heart failure, vascular disease, pneumonia, and dialysis; urbanization level; monthly income; and drug use. A stratified analysis was conducted to evaluate the effect of vaccination on age and sex (Table 2). All analyses were conducted using SAS software, Version 9.3 (SAS, Cary, NC, USA). A 2-tailed *P* value of <0.05 was considered significant. In sensitivity analyses, external adjustments improve the understanding of the effects of drugs and other covariates in epidemiological database studies [23]. Hence, in the sensitivity analysis, we made adjustments to examine the association of age, sex, CCI, comorbidities, and drug use with the incidence of ischemic stroke in different models. The models stratified by different seasons were adjusted for covariates in the main model and each additional covariate (Tables 3–5).

## CONCLUSIONS

Influenza vaccination might exert a dose-response effect against ischemic stroke in patients with AF who have risk factors for ischemic stroke by reducing the incidence of ischemic stroke, particularly in those aged 65–74 and ≥75 y.

## SUPPLEMENTARY MATERIALS TABLE



## Abbreviations

AF: atrial fibrillation
NHI: National Health Insurance
NHIRD: National Health Insurance Research Database
ICD-9-CM: International Classification of Diseases, Ninth Revision, Clinical Modification
ACEI: angiotensin-converting enzyme inhibitor
PS: propensity score
HR: hazard ratio
AD: Alzheimer disease
