# Supplementary material for: Influenza vaccination might reduce the risk of ischemic stroke in patients with atrial fibrillation: A population-based cohort study
Source: Oncotarget. 2017 Nov 9;8(68):112697–711. doi: 10.18632/oncotarget.22352 (PMC5762542; doi:10.18632/oncotarget.22352)
Supplement: Supplementary file 1 [file oncotarget-08-112697-s001.pdf]

Influenza vaccination might reduce the risk of ischemic stroke in patients with atrial fibrillation: A population-based cohort study

SUPPLEMENTARY MATERIALS

Supplementary Table 1: Candidate variables included in the logistic regression model

Candidate variables		Odds ratio	95% CI
**Age, ≥75 y**	1.283	1.206	1.367
Age, 55–74 y	0.439	0.422	0.491
Female sex	0.736	0.710	0.821
CCI, ≥3	0.873	0.821	0.942
CCI, 2	0.776	0.743	0.844
CCI, 1	0.567	0.526	0.610
Diabetes	1.435	1.402	1.550
Hypertension	1.689	1.602	1.788
Dyslipidemia	2.140	2.059	2.197
Congestive heart failure	1.310	1.271	1.352
Vascular disease	1.453	1.372	1.534
Pneumonia	1.191	1.142	1.243
Dialysis	1.223	1.162	1.294
Urban	0.982	0.924	1.044
Suburban	0.987	0.907	1.074
Income, ≥NT$33 301	0.838	0.763	0.920
Income, NT$21 000–33 300	0.956	0.867	1.056
Income, NT$1–21 000	0.930	0.840	1.030
Warfarin use	0.741	0.723	0.767
Statin use	1.343	1.252	1.431
Metformin use	1.666	1.573	1.762
Aspirin use	0.981	0.956	0.999
ACEI use	1.054	1.021	1.093

[+]CCI: Charlson comorbidity index
CI: confidence interval
